# Analysis and stress-test of the spatial accessibility to German radiation oncology centers

**DOI:** 10.1007/s00066-025-02435-7

**Published:** 2025-07-24

**Authors:** Christoph Straube, Daniel Medenwald, Tim Holthaus

**Affiliations:** 1 Klinik für Radioonkologie und Strahlentherapie, Klinikum Landshut, Robert-Koch-Str. 1, 84034 Landshut, Germany; 2 Klinik und Poliklinik für Strahlentherapie, Universitätsklinikum Halle (Saale), Ernst-Grube-Straße 40, 06120 Halle (Saale), Germany; 3 AG Versorgungsforschung, DEGRO, Berlin, Deutschland; 4 School of Architecture and Civil Engineering, University of Wuppertal, Pauluskirchstraße 7, 42285 Wuppertal, Germany

**Keywords:** Accessibility, Radiotherapy, Disaster, Resilience

## Abstract

**Background:**

Radiation therapy (RT) is a crucial component of cancer care. However, access to RT services varies significantly across regions. This study aims to assess and stress-test the geospatial distribution of radiation oncology centers (ROCs) in Germany.

**Methods:**

Geospatial analysis was used to calculate travel times and distances and to calculate regional RT demand. A regional network analysis was also conducted. Simulation of local ROC closure and recalculation was conducted to stress-test the remaining RT capacities, resulting in a redistribution network analysis (ReDNA).

**Results:**

While some regions have a higher concentration of ROCs than others, the average ROC usually serves a population of approximately 238,185 inhabitants. In urban regions, the average travel time to the nearest ROC was 7.9 min, compared to 11.4 min in rural regions. However, the heterogeneity increases when considering the second- or third-closest ROC, especially in rural regions. The regional ReDNA identified regions with high vulnerability to local ROC disruption. In these areas, the closure of a single ROC probably leads to significant increases in travel times for patients. Inhabitants of cities/towns in rural regions are specifically dependent on the serviceability of their ROC, with a relative increase in travel time by 171.9% if the currently nearest ROC is closed.

**Discussion:**

For the first time, this study investigates the supply of RT services on a national level. The spatial accessibility of RT services is analyzed by regional types as well as by federal states. The novel ReDNA approach allows regions with potential vulnerabilities to be identified, resulting in a framework for stress-testing RT supply on a national level.

**Conclusion:**

This research provides valuable insights into the spatial accessibility and vulnerability of radiation oncology services in Germany. Additionally, the methodology can also serve as a stress test and be applied to other regions and healthcare specialties to improve healthcare planning and patient outcomes.

**Supplementary Information:**

The online version of this article (10.1007/s00066-025-02435-7) contains supplementary material, which is available to authorized users.

## Introduction

Radiotherapy (RT) is a cornerstone of modern cancer care, and access to RT is linked to utilization and outcomes in cancer [1, 2].

Spatial heterogeneities in population density lead to an uneven distribution of radiation oncology (ROCs), with better access in urban than in rural regions [3]. This heterogeneity might be enhanced by the uneven distribution of privately insured patients, favoring urban regions, resulting in enhanced ROC density in metropolitan regions. The spatial distribution of radiotherapy centers in Germany, however, has not been studied in detail so far.

A lower density of ROCs in rural regions may result in an increased vulnerability of access to RT in rural regions in the case of a breakdown of a single ROC. Breakdown of ROCs became a realistic scenario during the COVID-19 pandemic, as impressively described by Buglione et al. [4]. Besides, natural disasters such as floods led to a shutdown of ROCs in Dresden in 2012 and Eschweiler in 2021 [5, 6]. Notably, the frequency of weather-related hazards is likely to increase with the ongoing aspects of climate change in Europe [7]. Beyond floods, other natural disasters like earthquakes and fires can be equally disruptive to the RT supply [8]. Today, cybersecurity threats also warrant consideration [9]. Additionally, changes in reimbursement and demography-driven reductions in the workforce of medical physicists, radiation therapists, and radiation oncologists could also limit the ROC supply. For example, the German physician registry shows an increase in the mean age of radiation oncologists from 49.4 years in 2013 to 51.4 years in 2022 [10]; data for radiation therapists and medical physicists are not available.

Considering natural disasters, dialysis services in the US have generated detailed plans to deal with local incidences [11, 12]. To the best of our knowledge, there is no analysis on the resilience of RT services available, nor do national plans to cope with disasters effecting ROCs exist. This study uses geodata analysis to investigate the accessibility and spatial vulnerability of radio-oncological care in Germany. Regional redistribution network analysis (ReDNA) is used to identify undersupply in the case of local ROC disruptions.

The proposed methodology can be used to optimize the spatial supply structure for radiation oncology in routine care. Knowledge of possible alternative structures also allows for adapted specifications for potential failure concepts, thereby helping to avoid unnecessary oversupply and accumulation in metropolitan regions while enhancing accessibility in rural regions.

## Methods

### Radiotherapy departments

A comprehensive strategy was developed to identify all operational ROCs in Germany. This involved searching national and international registries, conducting web searches, and contacting centers directly [13, 14].

This holistic approach yielded an initial list of 378 potential ROCs. Subsequent data cleaning excluded centers offering only specialized techniques (e.g., particle therapy, stereotactic radiosurgery, brachytherapy, orthovoltage therapy) or those identified as duplicates. This was done to focus on potential spatial redundancies in the availability of standard radiotherapy services across different regions of Germany. As a result, a final list of 337 operational linac-based facilities housing a total of 645 linear accelerators (linacs) in Germany was established (data current as of January 1, 2024).

Taking 645 linacs distributed across 337 ROCs yields an average of two linacs per ROC. Over one third (133) of German ROCs operate with a single linac, while a similar number (128) possess two linacs. Notably, 21 ROCs, primarily academic centers, are equipped with four or more linacs.

### Calculation of the average workload per linac

Data from the Bundesamt für Strahlenschutz indicated 201,615 malignant RT courses delivered in Germany during 2016 as well as 258,946 benign cases reported by 2016 [15]. Notably, there is a time gap between the used data sources: the most recent estimate of RT demand in Germany is from 2016, the census in Germany was in 2022, and the number of ROCs and linacs is based on data from January 2024. However, the German population remained almost stable from 2016 to 2022 (82.5 million inhabitants in 2022 as compared to 80.3 million in 2011); hence, the incidence rate of treatments is likely to be stable during this time period as well. We assumed an incidence of 320 benign and 250 malignant cases per 100,000 inhabitants. This approach does not reflect inequalities in age or regional differences in the cancer incidence.

Considering the national inventory of 645 linacs, this translates to an average of 312 malignant and 401 benign cases treated per linac annually. Notably, some of the malignant indications might have undergone treatment with special techniques such as radiosurgery, particle therapy, or brachytherapy in specialized centers not included in this analysis. Benign cases, on the other hand, are often treated with orthovoltage devices. Since only linacs have been included in this analysis, the number of malignant and benign courses treated at standard ROCs might be slightly lower.

Considering the simpler techniques often employed for benign cases, the potential for treatment with orthovoltage devices, and a typically lower number of treatment fractions, we assumed a workload equivalence of three benign cases to one malignant case, resulting in 136 “malignant-equivalent benign cases” (MEBC) [16–20].

This adjusted workload estimation suggests an average annual throughput of 445 malignant-equivalent cases (MEC; 312 malignant + 133 MEBCs) per linac. This aligns with data from the ESTRO HERO analysis (419 cases per linac in 2012) and the ESTRO QUARTZ project’s recommendation of 450 cases per linac [21–24].

### Geospatial analysis

To assess spatial accessibility to radiation therapy, car travel times and distances were calculated using Dijkstra’s algorithm. Certain relations showed shorter travel times of public transport than car travel. However, considering the average waiting times in public transport as well as the morning peak hours between 6.00 and 11.00 a.m., this holds true for only a limited number of relations [25]. Car travel time can therefore be used as a proxy for spatial accessibility at a national level, even though car ownership is lower in German metropolitan regions than in other regions [26]. This analysis considered 337 radiation locations and all 3.3 million inhabited 100‑m GeoGrids across Germany. Travel times do not include access, egress, and parking search times based on established guidelines, due to heterogeneous and individual parking situations at the residential and ROC locations [27]. Therefore, in this paper, travel times refers to car driving time unless otherwise specified. A flat rate consideration of access, egress, and parking search times can be compensated for by adding 5 min. Data on road networks and typical mid-morning weekday traffic speeds (validated against external sources and permanent traffic counting systems) originated from OpenStreetMap and the ADAC (German Automobile Club; [28]). Spatial analysis further enriched these data with official municipal keys (December 31, 2019) and RegioStaR7 spatial typologies [29].

The resulting data matrix with 3.3 million × 337 entries allows one to sort for the *n*-closest ROC for each inhabited GeoGrid [30]. As a first estimate, the catchment area per ROC was defined as the inhabited area with the shortest travelling time to a given ROC. Notably, this approach does not reflect the real catchment area per ROC, as areas with more than one ROC might have overlapping catchment areas. However, when used as a basis for a network analysis, the given matrix allows for resorting of the GeoGrids to the *n* + 1 and further ROCs.

### Regional redistribution network analysis

For each inhibited 100 m × 100 m GeoGrid, the travel distance and duration to every ROC in Germany were calculated as described above. To assess the impact of a specific ROC (ROC_A_) closure, residents previously assigned to ROC_A_ were reassigned to their new closest ROC (ROC_B_). Based on the initial number of patients at ROC_B_, the relative increase in patients resulting from closing ROC_A_ was calculated. Four levels of increase were anticipated, based on the effort needed in ROC_B_ to compensate for the increase in RT demand: (1) 0–10% increase, resulting in a minor change in workload; (2) 10–25% increase, resulting in a moderate change in workload compensable by an increase in working hours per day; (3) 25–50% increase, resulting in major change in workload compensable by an increase in workforce; and (4) > 50% increase, resulting in a substantial change in medical management, such as postponing patients, shorting fractionation, or reallocation to other ROCs.

Besides, a maximum of 800 MEC per linac and per year was deemed. This number was chosen in accordance with the OncoCert cancer center certificate in Germany, which requires (1) 800 cases and two linacs per ROC as well as (2) a 100% takeover of all cases from one linac to the remaining one in case of a breakdown of one linac. Given the average workload of 444 MECs per linac in a normal 8–10 h working day in Germany, 800 MEC per linac would be rendered possible with a working day of 14–15 h per day. This number is markedly above the European Society for Radiotherapy and Oncology (ESTRO) recommendations and within the real workload per linac in Brazil, where 14 h per day equals a normal working time per linac [31]. Notably, ROCs with a baseline workload of 800 or more MECs per linac per year are not able to compensate for a breakdown of neighboring ROCs without substantial changes in medical management.

### Manuscript writing

Figures were generated by RStudio Server 2023.09.0 Build 463 (R 4.3.1) as well as by QGis v3.16 (qgis.org). Tables were generated by Microsoft Excel 2021. The text was written in Microsoft Word 2021. To ensure linguistic accuracy as well as precise writing, the AI language model Gemini was employed, and a native speaker reviewed the text for correctness. From September to November 2024, individual sentences and paragraphs of the manuscript were analyzed and revised using Gemini. Neither were data analyzed, nor was any figure generated by AI applications.

## Results

### Travel distances and travel time to the distance closest ROC

**Fig. 1:**
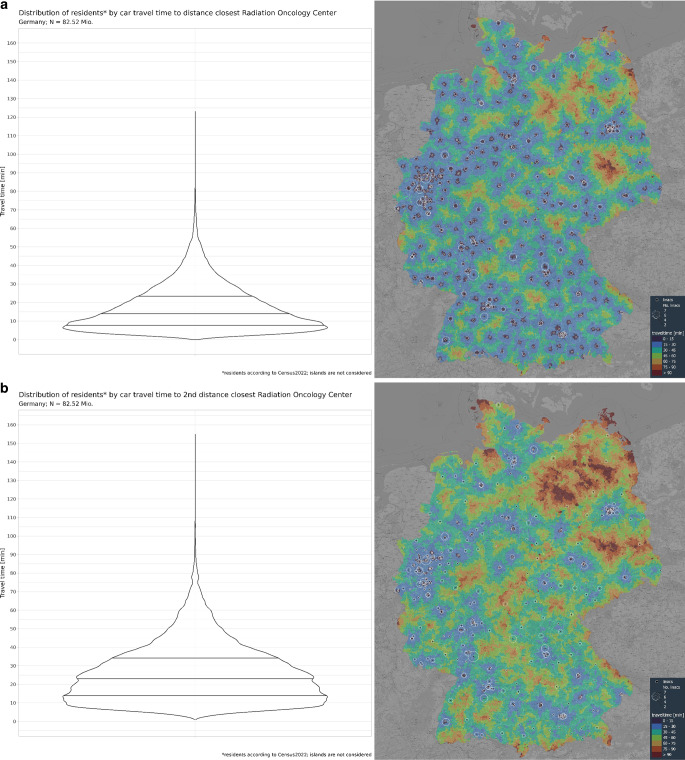
Violin plots (**a**, **c**) and maps (**b**, **d**) showing the travel time to the first (**a**, **c**) and second (**b**, **d**) distant-nearest radiation oncology center (ROC). Driving time for 06.00 to 11.00 a.m. on working days in 2017, including access, parking, and egress time according to RIN 2008. Colors on the maps represent travel times. Circles represent the number of linacs per ROC. Map background from ©OpenStreetMap contributors

The travel time averaged over the entire area of Germany to the distance closest ROC with was 17.1 min. The median travel time was 14.1 min. 95% of the German population will need to travel equal or less than 40.1 min to reach the distant closest ROC (Fig. 1). This corresponds to travelling in average 15.5 km, median 11.1 km. 95% of the inhabitants live within 42.7 km route distance of the nearest ROC (Table 1, Figure Supp_1).

**Table 1:** Travel distances to the first and second closest ROC, either selected by the fastest track (time-closest) or the shortest track (distance-closest).

**Travel distances and travel time to the 1st and 2nd distance-closest ROC**						
	*mean distance (km)*	*mean time (minutes)*	*median distance (km)*	*median time (minutes)*	*p95% distance (km)*	*p95% time (minutes)*
1st	15.5	17.1	11.1	14.1	42.7	40.1
2nd	27.2	25.9	23.9	23.2	63.6	55.6
**Travel distances and travel time to the 1st and 2nd time-closest ROC**						
	*mean distance (km)*	*mean time (minutes)*	*median distance (km)*	*median time (minutes)*	*p95% distance (km)*	*p95% time (minutes)*
1st	16.7	16.7	13.9	13.9	39.4	39.4
2nd	28.1	25.2	24.3	23.0	66.9	52.3

*ROC* radiation oncology center, *p95%* the time or distance, respectively, within which 95% of the population reach the nearest ROC (either first or second closest)

**Table 2:** Number of inhabitants per radiation oncology center (ROC) and per linac, either by federal state or by catchment area defined by the duration of travel or the distance of travel criteria. Number of inhabitants as by Census 2022.

German federal state	Residents within the n1 catchment area by duration criteria	Residents within the n1 catchment area by distance criteria	Inhabitants	Linacs	ROCs	Inhabitants per linac (duration criteria)	Inhabitants per linac (distance criteria)	Inhabitants per ROC (duration criteria)	Inhabitants per ROC (distance criteria)	Linacs/ROC
Baden-Württemberg	10,941,682	11,082,860	11,104,040	82	37	133,435	135,157	295,721	299,537	2.22
Bayern	13,422,580	13,312,024	13,038,724	105	59	127,834	126,781	227,501	225,628	1.78
Berlin	4,375,259	4,494,888	3,596,999	34	16	128,684	132,203	273,454	280,931	2.13
Brandenburg	1,619,745	1,527,369	2,534,075	13	7	124,596	117,490	231,392	218,196	1.86
Bremen	1,105,177	1,067,984	693,204	9	4	122,797	118,665	276,294	266,996	2.25
Hamburg	2,663,108	2,724,993	1,808,846	18	7	147,295	151,389	380,444	389,285	2.57
Hessen	6,058,437	6,025,320	6,207,278	33	17	183,589	182,585	356,379	354,431	1.94
Mecklenburg-Vorpommern	1,535,275	1,525,489	1,570,817	11	4	139,570	138,681	383,819	381,372	2.75
Niedersachsen	7,342,757	7,327,690	7,943,265	49	29	149,852	149,545	253,199	252,679	1.69
Nordrhein-Westfalen	18,301,547	18,335,362	17,890,489	168	94	108,938	109,139	194,697	195,057	1.79
Rhineland-Pfalz	3,347,604	3,290,026	4,094,169	30	17	111,587	109,668	196,918	193,531	1.76
Saarland	1,065,090	1,055,880	1,006,864	11	5	96,826	95,989	213,018	211,176	2.20
Sachsen	4,040,991	3,992,795	4,038,131	23	11	175,695	173,600	367,363	362,981	2.09
Sachsen-Anhalt	1,897,072	1,885,372	2,146,443	15	7	126,471	125,691	271,010	269,339	2.14
Schleswig-Holstein	2,581,326	2,512,033	2,927,542	22	12	117,333	114,183	215,111	209,336	1.83
Thüringen	2,217,891	2,355,456	2,110,396	22	11	100,813	107,066	201,626	214,132	2.00

By not considering the distance-closest but rather the time-closest reachable ROC, the average travel time reduces to 16.7 min, with 95% of the population reaching an ROC within 39.4 min.

### Travel distances to the second-closest ROC

For the whole area of Germany, the average travel time to the second distance-closest ROC with a car was 27.2 min. The median travel time was 23.9 min; 95% of the German population will need to travel equal to or less than 63.6 min to reach the second distant-closest ROC (Fig. 1).

When considering the second time-closest ROC, the average travel time was 25.2 min, with 95% of the population reaching a ROC within 52.3 min (Fig. 1).

### Spatial accessibility in urban vs. rural regions

For the following analysis, the RegioStaR7 spatial typologies (in the following RS/region) of the Federal Ministry for Digital and Transport (BMDV) are used. These regions address transport infrastructure and mobility behavior [32]. The ROCs accumulate in urban regions, with 230 ROCs (68%; RS 71–74) where 52.7 million inhabitants (63%) live; 107 ROCs (32%) are in rural regions, mostly in towns and cities of rural regions (29.75 million inhabitants live in rural regions; 37%; RS 75–77).

The average travel time and distances to the time-closest ROC in the urban regions (RS 71–74) is 7.9–21.7 min or 4.9–22.0 km (median 7.2 to 20.9 min or 4.1–21.2 km), compared to an average of 11.4–29.3 min or 14.9–31.3 km (median 15.3–27.9 min or 13.6–29.1 km) in rural regions (RS 75–77; Supplemental Table 1, Fig. 3).

The heterogeneity increases by considering the second- or third-closest ROC. The average travel times and distances in urban regions are 11.0–29.5 min or 7.3–33.7 km to the second-closest (median 10.3 to 27.3 min, 6.3 to 29.9 km) and 13.8–36.2 min or 9.8–44.6 km to the third-closest ROC (median 12.8 to 34.2 min, 8.3 to 40.0 km). This represents an increase in travel time from the first- to second-closest ROC by 48%/125%/40.7%/35.9% and from the first- to third-closest ROC by 74.6%/217.5%/68%/66.8% for RS 71/72/73/74, respectively.

**Fig. 2:**
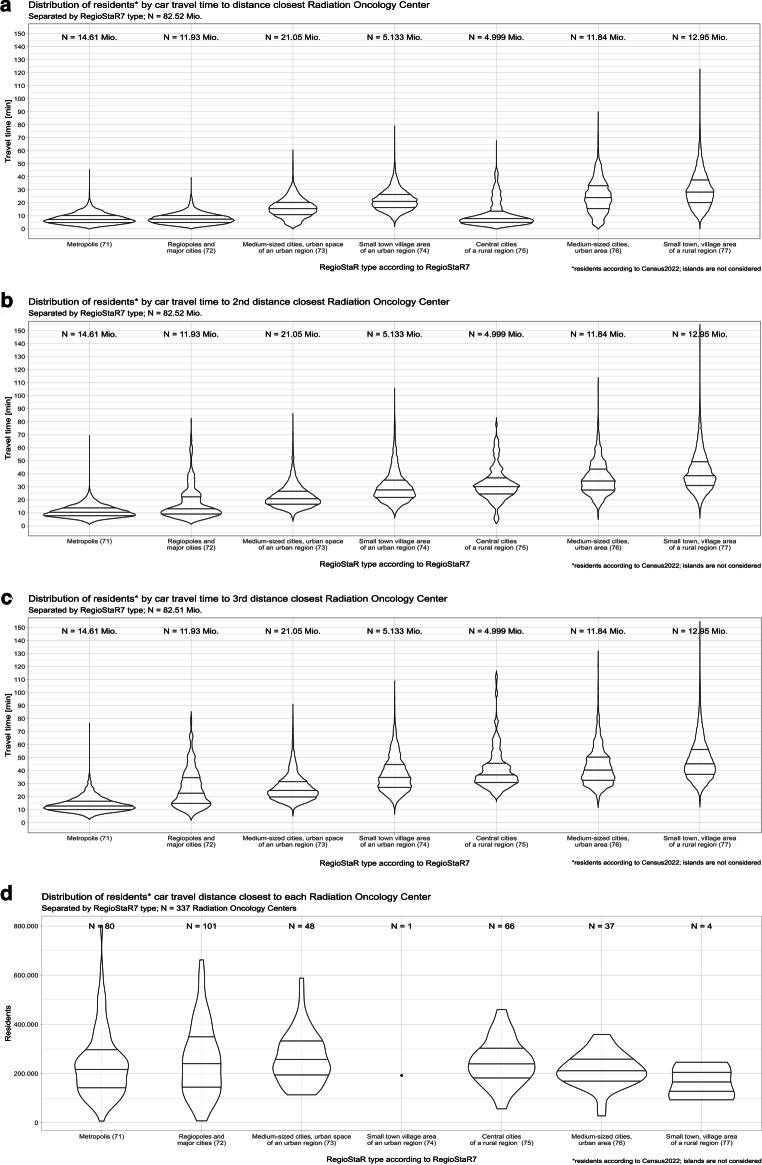
Violin plots showing the distribution of residents and travel durations of inhabitants per regional structure by car travel distance to the first (**a**), second (**b**), and third (**c**) distance-closest radiation oncology center. The width of the plot corelates to the number of residents travelling for a given duration. (**d**) Shows the number of residents as defined by the distance criteria

In rural regions, the increase in travel time and distance from the first- to second- or third-closest ROC is steeper. The average travel times and distances in the rural regions (RS 75–77) are 11.4–29.3 min or 9.8–31.3 km for the first-closest ROC 31–40.4 min or 39.7–48.7 km to the second-closest (median 29.9 to 37.9 min, 36.5 to 45.7 km) and 38.6–46.8 min or 52.5–59.5 km to the third-closest ROC (median 36.6 to 44.4 min, 47.7 to 55.8 km). This represents an increase in travel time from the first- to the second-closest ROC by 171.9%/44.6%/37.9% and from the first- to the third-closest ROC by 238.6%/71.7%/59.7% for RS 75/76/77, respectively (Fig. 2 and supplemental Fig. 2).

Spatial analysis of the ROC distribution also allows quantification of the population density surrounding each ROC. The average ROC serves a population of approximately 238,185 inhabitants (median: 222,995; range: 6598–738,901). The median number of inhabitants living closest to an ROC is higher in regional cities (RS 72) and medium-sized cities (RS73), but the difference is relatively small.

### Spatial accessibility by federal state

The distribution and size of ROCs appear to be uneven in Germany. ROCs in Mecklenburg-Vorpommern have 2.75 linacs per ROC and care for about 380,000 residents per ROC. ROCs in Niedersachsen are smaller, with only 1.7 linacs per ROC, caring for about 250,000 residents. The number of residents per linac ranges from about 96,000 (linacs in Thüringen) to about 182,000 residents per linac in Hessen (Table 2). Notably, ROCs from one federal state might care for patients from a neighboring state. This is particularly interesting in Brandenburg with Berlin in its center (about 130,00 residents per linac) and, vice versa, for Bremen surrounded by Niedersachsen (about 150,000 inhabitants in Niedersachsen vs. about 120,000 residents per linac in Bremen; Supplemental table 2). ROCs in one federal state do attract patients from neighboring federal states.

**Fig. 3:**
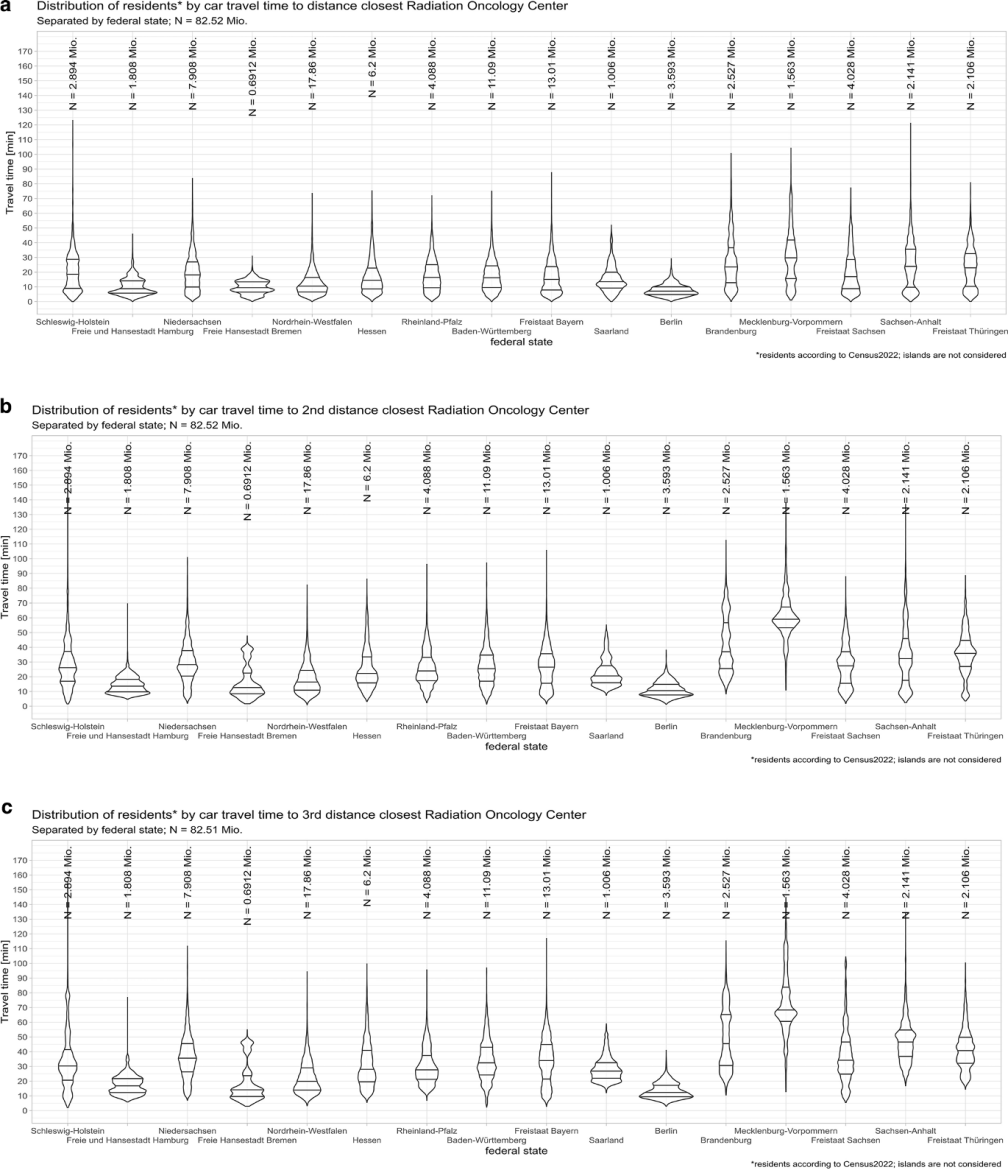
Violin plots showing the distribution of residents and travel duration of inhabitants per federal state, by car travel distance to the first (**a**), second (**b**), and third (**c**) distance-closest ROC

Inhabitants of Berlin have the shortest travel time to the time-closest ROC, with an average of 7.7 min (median 7.1 min); 95% of inhabitants of Berlin reach their time-closest ROC within 14.6 min. In contrast, inhabitants of Mecklenburg-Vorpommern travel 30.3 min on average (median 29.6 min); 95% of inhabitants reach their closest ROC within 62.6 min (Fig. 3 and supplemental Fig. 3).

Traveling to the second- or third-closest ROC is correlated with large discrepancies between the federal states. Those states with a more urban structure (e.g., North Rhine-Westphalia, Berlin), where the most carless individuals live and public transportation is generally well developed, experience only negligible differences in driving times due to high ROC density, while predominantly rural states such as Mecklenburg-Vorpommern are effected by a substantial increase in the duration of travel.

### Estimation of the vulnerability of RT services to ROC closure

**Fig. 4:**
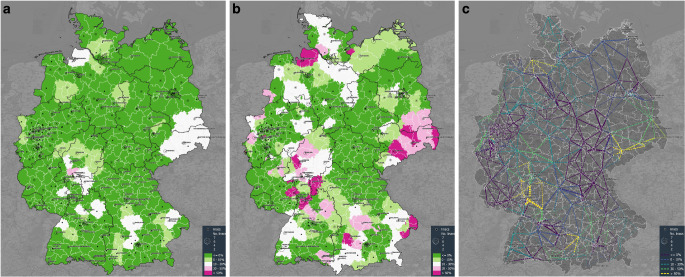
Results from the network analysis. Each radiation oncology center (ROC) was iteratively closed; all patients assigned to this ROC were redistributed to the second-nearest ROC by travel time criteria. **a** Capacity exceedance of the ROC cluster before closing of the kernel ROC identified by catchment area using a color scale; **b** capacity exceedance of the ROC cluster after closing of the kernel ROC; **c** redistribution network. The color of the width of the line correlates to the closet ROC, while the color scale depicts the capacity exceedance of the ROC cluster

The ad hoc capacity of regions is mostly homogenous distributed, with only very view regions exceeding the anticipated threshold of 800 MEC per linac (Fig. 4). Notably, the presence of orthovoltage devices, particle treatments, or a less-frequent use of RT for benign indications could limit the workload per linac in these regions and, hence, result in a well-compensated workload.

Simulating the capacity with each ROC closed reveals a solid redundancy of radiotherapy services in Germany. Especially regions with previously limited capacity and regions around very large ROCs are at risk of developing a regionally limited RT undersupply in the case of a local ROC breakdown.

## Discussion

This study provides a comprehensive analysis of the spatial accessibility of radiation oncology and radiotherapy services in Germany. Information about the anticipated capacities allowed us to visualize regional shortages as well as vulnerability of ROC services. The methodology of systematically investigating the redistribution of catchment areas and capacities (ReDNA) used in this manuscript is unique and has never been described for radiation oncology or other medical specialties before. The methodology allows one to stress-test the national supply of RT services for local ROC failure. It appears to be generalizable to other countries or specialties and easily allows the addition of additional information, if available.

### Spatial accessibility

Several groups have investigated the accessibility of ROCs. The concept of accessibility includes limitations to access due to economic, social, or institutional barriers. Many of these barriers do not apply to Germany, as the general social insurance system covers not only the treatment costs, but also transportation. Hence, in Germany, the spatial accessibility largely equals the accessibility of ROCs.

A recently published systematic review of 168 publications described an expected high heterogeneity, depending primarily on the economic background and the degree of urbanization of the region under study. Accordingly, mean travel distances in urban regions in the US ranged between 9.26 and 16 miles (14.9–25.7 km), while distances in rural areas were significantly longer (40.2–59 miles, 64.7–95 km) [33]. Furthermore, travel distances are an important factor in calculating the environmental impact of RT, as recently shown in an extensive life cycle analysis of four large academic centers in the US [34].

The locations of ROCs are distributed in relation to resident density. Hence, metropolitan regions offer a high density of ROC options to their residents, allowing free choice of the treating ROC with minimal additional effort. On the other hand, residents of rural regions or federal states with a high proportion of rural regions are more likely to be treated at the closest ROC, as the effort to reach competing ROCs might be too large. Importantly, Germany allows treatment in any ROC, not restricting patients to their federal state or residence.

With regard to the German oncologic infrastructure, the available literature is sparse. An article from 2021 investigated the spatial accessibility to specialist breast cancer treatment in Bavaria. The article reported a mean travel time of 13 min (0–73 min) in urban and 21 min (0–79 min) in rural areas [35]. Furthermore, a recent article investigated the real-life catchment area of four ROCs based on real data from 4198 treated breast cancer patients. The average travel distance was 37.2 km per fraction or 18.6 km per single trip. These results fit well with our data, with mean distances of 15.5 km (distance closest ROC) and 16.7 km (time-closest ROC), especially as patients might chose the second- or third-closest ROC due to personal decisions [36]. Considering a wide range of travel times and distances in an international context, Germany provides one of the narrowest networks of RT services worldwide [33]. With as few as 238,185 inhabitants per ROC in Germany (4.2 ROC per million), only Switzerland has a higher density of ROCs in Europe (4.7 ROCs per million inhabitants) [22]. With 1.9 linacs per ROC, the size of German ROCs is below the European average (median 2.6), only Bulgaria (0.9), Belarus (1.5), and Switzerland (1.6) have fewer Linacs per ROC [22].

Investigating travelling times to the second- or third-closest or more distant ROC allows the accessibility of a second opinion to be investigated for the first time. We describe important differences in the difficulties of finding a second specialist especially in rural regions, while inhabitants of metropolitan regions can choose between several ROCs with only negligible travel distances. Especially carless inhabitants in rural regions are disproportionately impacted by extended travel times when the nearest ROC is unavailable. As the proportion of inhabitants per ROC is almost equal in between urban and rural regions, larger travel distances are an inevitable consequence of the uneven distribution of the population. Whether this is associated with an effect on outcomes is unknown.

### Vulnerability

The current work is the first investigation not only for the closest facility but also for the second to each subsequently next ROC. This enabled us to stress-test the national ROC infrastructure by investigating the effect of local ROC failure on the spatial accessibility and workload of the remaining ROCs. This allows us to quantify the local vulnerability of the radiation oncology supply. Thereby, we identified regions with a relevant vulnerability to local incidences affecting ROCs. Modeling ROC capacities by regional networks enhances the robustness of this approach.

Another common method to investigate catchment areas is the “two-step floating catchment area method” [37]. This methodology allows one to incorporate multiple additional information, although it is not designed to model elimination of centers and subsequently re-evaluate the accessibility. The “three-step floating catchment area” is designed to overcome this limitation, however, it is usually applied only at the level of cities or limited regions [38]. Furthermore, the demand for services decays in predefined travel-time levels, which inadequately reflects the demand for treatment of acute life-threatening diseases, such as cancer.

In the absence of more detailed information on structural and personal infrastructure, our proposed methodology provides the most accurate approximation for estimating ROC supply and its vulnerability. Surprisingly, there is no research on this topic available. Related work investigates the search for the most-effective localization of new facilities. A landmark study from Brazil proposed a shortage index based on regional incidences and ROC supply. This approach is a suitable approach to deal with severe undersupply and provides information on potential oversupply. A shortcoming is the lack of accessibility information, as only regions and states were used as a basis for the score [39].

In summary, our data show a high level of resilience in most regions of Germany, yet at the cost of a significant increase in travel times of up to an additional 19.6 min (inhabitants of cities in rural regions, mean travel time, relative increase 171.9%) in some regions. The very few regions with an above-average workload per linac or with limited resilience are recommended to undertake an in-depth analysis of the current workload, workforce, and level of redundancies of the involved ROCs. Potentially, the affected ROCs have already implemented hypofractionation to a larger extent, use orthovoltage devices for benign disorders, or offer specialized linacs for SBRT. All these options free capacities from linacs, enabling treatment of more malignant cases. Consequently, former analyses of linac capacities, such as ESTRO HERO, ESTRO QUARTS, or the MALTHUS project—all of them showing an ideal catchment of about 120,000 residents per linac—need to be adapted to modern techniques and treatment concepts [21–24, 40].

Notably, this work focused on physical and local events affecting ROCs. Networks for RT planning, whose failure would impact multiple ROCs, were not considered. Furthermore, ROC breakdown was assumed to result in redistribution to the second-closest ROC. This is not necessarily the case, as commercial ROCs might redistribute patients to ROCs of their own network rather than to the second-closest ROC. Also, patient preferences are not included in the model. Lastly, the proposed model does not include changes of traffic infrastructure, namely roads or bridges. These changes could be easily integrated.

### Mitigation

As shown in this report, there is excellent spatial accessibility and resilience available in Germany. However, the generation of resilience is based on three cornerstones: the ability to redistribute and absorb the unsatisfied demand, the ability to restore disturbed infrastructure, and implementation of adaptations of in order to increase the resilience to future disruptions [41]. This report focusses on a potential redistribution of patients as a measure of resilience. In most regions of Germany, the second-closest neighboring ROCs can take over patients in case of a local disruption. If this is not possible, three modes of action are recommended:

Patients who are re-referred during their course of treatment currently have limited options for continuing their treatment. Contracts between ROCs, made to ensure resilience, often dictate specific ROCs for redistribution, which may not be the nearest ones. Consequently, patients could experience significant distress and could decide against continuing treatment if travel distances become too long [1, 42, 43]. Technical solutions, such as telemedicine, could ease the transfer of patient data and offer alternatives to the current contract-based resilience system [39].

Secondly, areas with limited redistribution capacities could increase local supply by reducing the mesh size of the local network; hence, by adding new ROCs. This ensures continuous treatment without changing fractionation regimens, indications, or techniques. However, it is costly and requires significant preparation time. Additionally, demographic changes are leading to a reduction in the number of radiation oncologists, thus creating challenges for maintaining the service.

Lastly, an increase in the proportion of hypo- and ultrahypofractionated regimens would reduce the number of patients per day per linac significantly [44]. As a positive side effect, hypofractionated schedules are associated with higher convenience for patients and also reduce the CO_2_ emissions associated with the total travel distance [36, 45, 46]. Besides these obvious positive effects, an increase in the total number of patients per linac as well as an increase in the complexity of treatments might result in increased wear and tear of the linacs, with a resulting requirement for maintenance and an increase in down-times [47]. Notably, this approach is very cost effective and requires only short times for implementation.

### Limitations

Our study is subject to several limitations. First, the assignment to the nearest ROC is based on travel time proximity, which may not accurately reflect the actual travel time due to potential capacity constraints of a given ROC. Hence, patients might be forced to travel to the second- or third-closest ROC. We used a network-based approach focusing on regional capacities to mitigate this limitation.

Second, patient preferences could not be considered. Especially in rural regions, when individual thresholds of travel distances are exceeded, this could lead to decisions against RT [1, 48]. On the other hand, patients in metropolitan regions might choose to be treated at the second- or third-closest ROC, as the time penalty between competing options is low.

Third, our analysis was limited to data from Germany, and cross-border patients may have distorted the picture. Given potential direction-specific reliefs or disincentives, local capacities could be over- or underestimated in these areas. Besides, islands were excluded from the study. However, as only very few inhabitants in Germany live on islands, the influence of this limitation is likely to be limited.

Fourth, we modelled the radiotherapy demand based on health care reimbursement data from 2016, which represent the real incidence of RT in Germany in 2016. Given the easy accessibility to RT due to the high level of insurance coverage as well as the high density of ROCs in Germany, the number of non-treated indications is deemed to be low. Another approach would be to model radiotherapy demand based on cancer incidences, as done for calculating the current and 2050 global and national workforce demand of RT [49]. We decided against the latter approach, as more assumptions would be needed to model RT demand. Furthermore, the granularity of openly available data in Germany is currently too coarse to be included in our analysis.

Fifth, we only considered car traffic, not train/subway or bicycle routes. Especially metropolitan regions have a highly developed commuting system which might modify the catchment area.

Finally, our capacity estimation is based on 40 h/week and frequent use of conventional fractionation. Longer service times per day or week can significantly increase the capacity, and centers in Brazil have documented treatment of more than 900 patients per linac annually [31]. Along these lines, the assumption of three benign cases equaling the workload of one malignant case was chosen in a best guess-manner. A higher ratio would result in higher capacities, while a lower ratio would result in an increased workload. Similarly, a higher proportion of hypofractionation can increase the patients treated per machine. On the other hand, centers with low personnel resources might not be able to further increase their throughput over baseline. Therefore, regions exceeding the assumed maximum number of cases of 800 patients per linac should be reviewed thoroughly in lieu of these limitations. Another factor affecting the estimated capacities is the real disease burden in a given area. Our analysis assumes an equal distribution of disease burden based on the number of inhabitants. However, the age distribution is uneven, with the highest average age in the eastern federal states of Germany. Hence, the workload in areas with an over-average age is at risk of underestimation.

## Conclusion

Analysis of regional ROC capacities based on travel times, travel distances, and regional redistribution networks (ReDNA) allowed us to identify regions with under-average ROC redundancies. This allows us to stress-test ROC capacities on a national level. The given approach is generalizable to other specialties and countries and allows inclusion of more parameters, if necessary. The presented methodology enables providers to review local capacities with real-world data.

## Supplementary Information

**Supplemental Table 1:** Travel times and distances to the closest and second- and third-closest ROC, either by selecting the time- (upper table) or the distance-closest ROC (lower table).

**Fig. 1_supplement**: Violin plots (A, C) and maps (B, D) showing the travel distance to the first (A, C) and second (B, D) distant-nearest ROC. Driving time for 06.00 to 11.00 a.m. on working days in 2017, including access, parking, and egress time according to RIN 2008. The width of the plot represents the number of residents traveling a specific distance to reach the destination. Colors on the maps represent travel times. Circles represent the number of linacs per ROC. Map background from ©OpenStreetMap contributors.

**Fig. 2_supplement: **Violin plots showing the distribution of residents and travel distance of inhabitants per regional structure, by car travel distance to the first, second, and third distance-closest ROC. The width of the plot corelates to the number of residents travelling a given distance.

**Fig. 3_supplement:** Violin plots showing the distances of residents and travel duration of inhabitants per federal states, by car travel distance to the first (A), second (B), and third (C) distance-closest ROC.

## Data Availability

All analyzed data are freely available at 10.57806/0d2gv0by.
