# Correction to: Analysis and stress-test of the spatial accessibility to German radiation oncology centers

**DOI:** 10.1007/s00066-025-02464-2

**Published:** 2025-09-09

**Authors:** Christoph Straube, Daniel Medenwald, Tim Holthaus

**Affiliations:** 1 Klinik für Radioonkologie und Strahlentherapie, Klinikum Landshut, Robert-Koch-Str. 1, 84034 Landshut, Germany; 2 Klinik und Poliklinik für Strahlentherapie, Universitätsklinikum Halle (Saale), Ernst-Grube-Straße 40, 06120 Halle (Saale), Germany; 3 AG Versorgungsforschung, DEGRO, Berlin, Deutschland; 4 School of Architecture and Civil Engineering, University of Wuppertal, Pauluskirchstraße 7, 42285 Wuppertal, Germany

**Correction to:**

**Strahlenther Onkol 2025**

10.1007/s00066-025-02435-7

In Table 2 of this article, in column “Inhabitants per linac (Duration criteria)”, the value for “Hamburg” was incorrectly given as “1472,950” and should have read “147,295”.

The original article has been corrected.

