# Supplementary figures and images for: Analysis and stress-test of the spatial accessibility to German radiation oncology centers
Source: Strahlenther Onkol. 2025 Jul 24;202(4):405–17. doi: 10.1007/s00066-025-02435-7 (PMC12999773; doi:10.1007/s00066-025-02435-7)

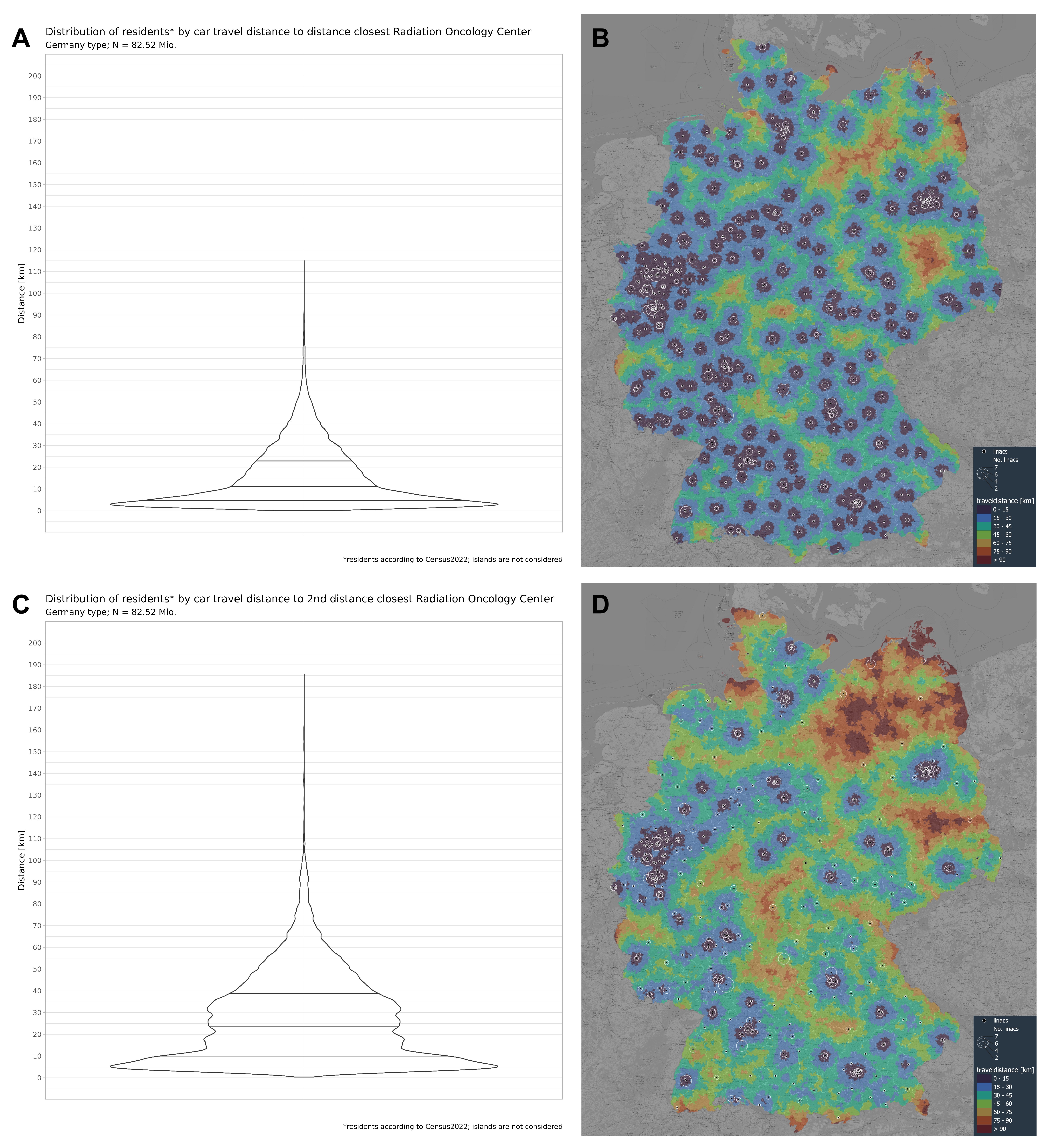

Supplement: Supplementary file 2 — Fig. 1_supplement: Violin plots (A, C) and maps (B, D) showing the travel distance to the first (A, C) and second (B, D) distant-nearest ROC. Driving time for 06.00 to 11.00 a.m. on working days in 2017, including access, parking, and egress time according to RIN 2008. The width of the plot represents the number of residents traveling a specific distance to reach the destination. Colors on the maps represent travel times. Circles represent the number of linacs per ROC. Map background from ©OpenStreetMap contributors. [file 66_2025_2435_MOESM2_ESM.jpg]

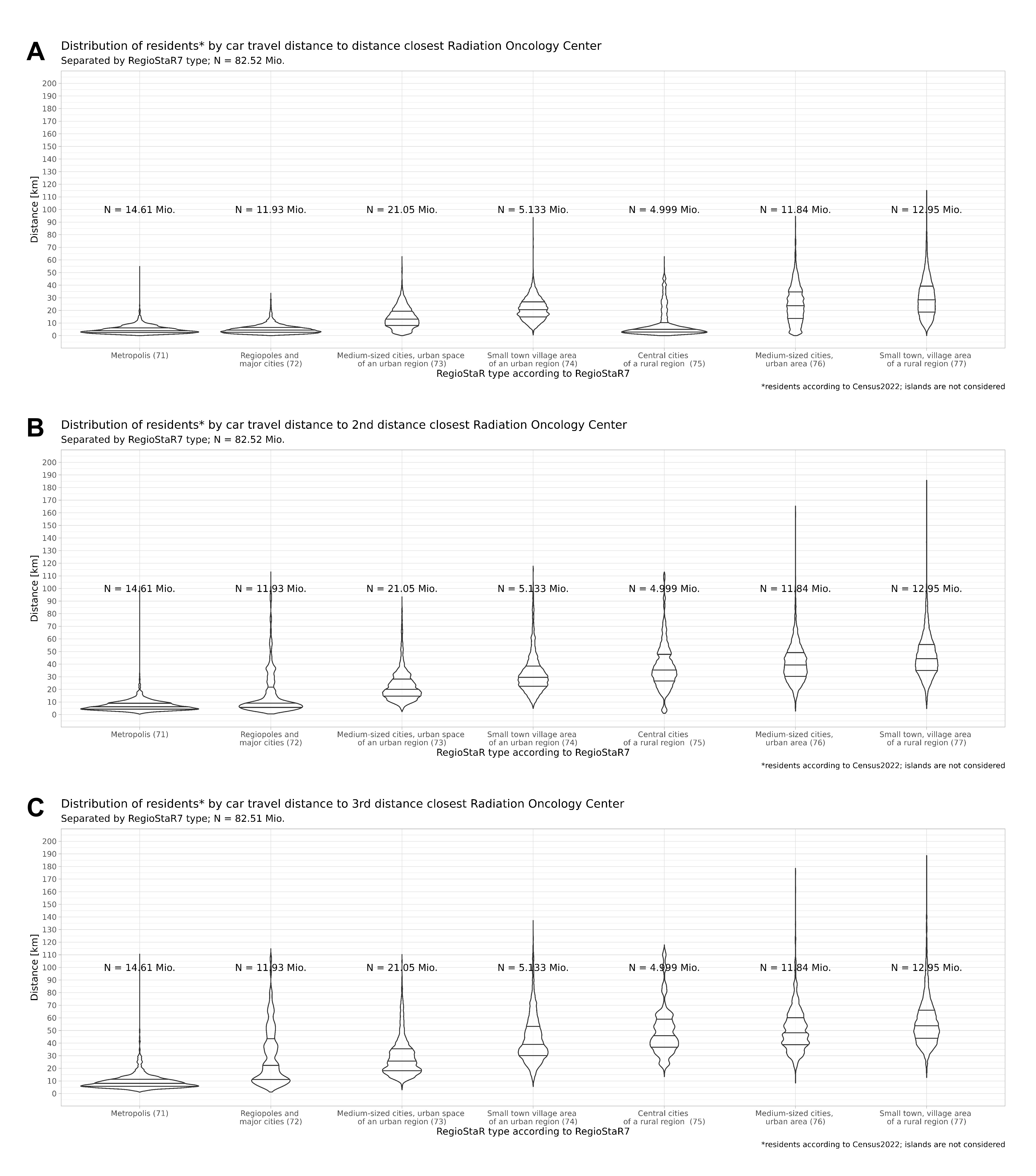

Supplement: Supplementary file 3 — Fig. 2_supplement: Violin plots showing the distribution of residents and travel distance of inhabitants per regional structure, by car travel distance to the first, second, and third distance-closest ROC. The width of the plot corelates to the number of residents travelling a given distance. [file 66_2025_2435_MOESM3_ESM.jpg]

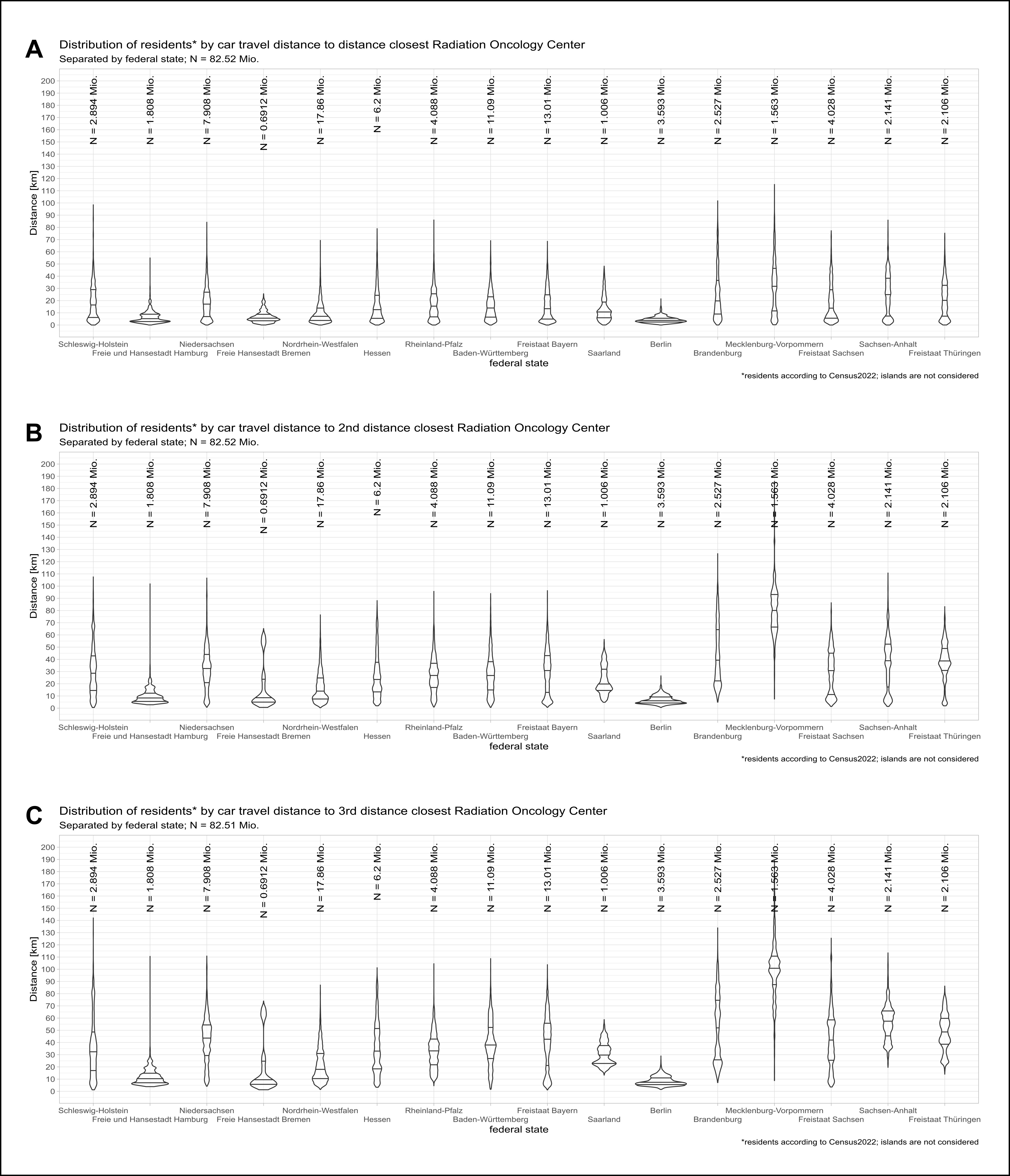

Supplement: Supplementary file 4 — Fig. 3_supplement: Violin plots showing the distances of residents and travel duration of inhabitants per federal states, by car travel distance to the first (A), second (B), and third (C) distance-closest ROC. [file 66_2025_2435_MOESM4_ESM.jpg]
